# Large language models and the perils of their hallucinations

**DOI:** 10.1186/s13054-023-04393-x

**Published:** 2023-03-21

**Authors:** Razvan Azamfirei, Sapna R. Kudchadkar, James Fackler

**Affiliations:** 1 Department of Anesthesiology and Critical Care Medicine, Johns Hopkins University School of Medicine, Baltimore, MD USA; 2 Department of Pediatrics, Johns Hopkins University School of Medicine, Baltimore, MD USA

## To the Editor,

We read with great interest the paper by Salvagno et al. [1] As they masterfully stated, “ChatGPT work should not be used as a replacement for human judgment, and the output should always be reviewed by experts before being used in any critical decision-making or application.” As is often the case in critical care, new technologies and apparent breakthroughs are often touted as game-changers. However, the truth usually emerges the next day when the confetti has settled, and we have to clean up the sticky mess left by gallons of printed ink mixed with our hopeful wishes.

Salvagno et al. present a ChatGPT-generated summary of three studies. As they noted, the summary was believable, albeit generic and sparse in the details. The glaring problem is that it’s completely fabricated. ChatGPT cannot access the internet, and its training dataset stops in September 2021; it has no reference to any studies published in 2023 [2]. In fact, one of the trials included in the summary, Belohlavek et al. [3], showed no improvement in functional neurological outcomes, contradicting ChatGPT’s summary.

We must understand one particular aspect of large language models, which is gracefully termed as “hallucinations”, though “fabricating information” may be more accurate [4]. In the case of the ChatGPT summary, we are only reading a generic summary of an average study with a given title.

The following is an excerpt from a “self-reflective” ChatGPT conversation.

“Q: Briefly, what is a GPT?

A: It is a type of deep learning algorithm that is designed to generate human-like text. […] Once trained, GPT models can be used to generate new text that is coherent and follows the patterns and styles of the original training data.

Q: Would it be fair, then, to say that GPT models, such as yourself, use a probabilistic approach to generate text that looks like previous text that was written?

A: Yes, that is a fair statement. […] When given a prompt, the model generates text by predicting the most likely word or phrase to follow based on the patterns it has learned from the training data.

Q: When I ask you to generate new text, are you coming up with the ideas yourself or just repackaging previously written works?

A: […] I do not come up with the ideas myself. Instead, I generate text based on patterns and relationships within my training data. This means that I am essentially repackaging previously written works, based on what I believe to be the most probable continuation of the given input text.

Q: Is there any filter of correctness applied to the output?

A: As an AI language model, I do not have a built-in filter to determine the correctness of my output.”

When﻿ the model had no answer, we received whatever looked like the most probable summary of the study—a hallucination.

Of course, we will improve our ability to integrate real-time information and reduce the rate of “hallucinations”, just as the nascent field of prompt engineering evolves. Running the exact prompt used by the authors now results in a response recognizing the knowledge gap. The broader point is less about whether we should use ChatGPT, and more about whether we are using it with a full understanding of its strengths and limitations [5].

We commend the authors for their exploration of ChatGPT and some associated important ethical issues. Foremost, however, it is important to reiterate that because Chat GPT now has no access to the article it was asked to interpret, it was given an impossible task. Our goal is simply to emphasize that, whether it’s a new language model, an innovative monitoring technology, or a novel biomarker, we must be aware of our tools' limitations. We hope that as these technologies evolve, they respond, as did Robot Model B-9 in the 1960’s television show *Lost in Space,* with “that does not compute” before spewing what it must know is a hallucination.

We offer this analogy as a conclusion. Imagine a self-driving system trained to safely navigate a car on public roadways; would we place the same system in a rocket, asking it to navigate us to low earth orbit? Likely not. The tasks seem similar, navigating, but are completely different. We can build systems to take us to Earth’s orbit, just how we’ll build systems to accurately summarize scientific articles. Our only hope is that we know whether our rocket is taking us to Kansas or the International Space Station *before* strapping ourselves to it. To again quote Robot Model B-9, “Danger”.

## Data Availability

Not applicable.
